# Evaluating the Quality Surface Performance of Additive Manufacturing Systems: Methodology and a Material Jetting Case Study

**DOI:** 10.3390/ma12060995

**Published:** 2019-03-26

**Authors:** Razvan Udroiu, Ion Cristian Braga, Anisor Nedelcu

**Affiliations:** Department of Manufacturing Engineering, Transilvania University of Brasov, 29 Eroilor Boulevard, 500036 Brasov, Romania; braga.ion.cristian@unitbv.ro (I.C.B.); a.nedelcu@unitbv.ro (A.N.)

**Keywords:** additive manufacturing, methodology, artifact configurations, design of experiments, surface roughness, material jetting, polymer additive manufacturing

## Abstract

The performance characterization of the manufacturing processes for additive manufacturing (AM) systems is a significant task for their standardization and implementation in the industry. Also, there is a large diversity of materials used in different AM processes. In the present paper, a methodology is proposed to evaluate, in different directions, the performance of an AM process and material characterization in terms of surface quality. This methodology consists of eight steps, based on a new surface inspection artifact and basic artifact orientations. The proposed artifact with several design configurations fits different AM systems sizes and meets the needs of customers. The effects of main factors on the surface roughness of up-facing platens of the artifacts are investigated using the statistical design of experiments. The proposed methodology is validated by a case study focused on PolyJet material jetting technology. Samples are manufactured of photopolymer resins and post-processed. Three factors (i.e., artifact orientation, platen orientation, and finish type) are considered for the investigation. The case study results show that the platen orientation, finish type, and their interaction have a significant influence on the surface roughness (Ra). The best Ra roughness results were obtained for the glossy finish type in the range of 0.5–4 μm.

## 1. Introduction

### 1.1. Additive Manufacturing Standardizations

Additive manufacturing (AM) [1] techniques garner much interest within many fields, such as aerospace, automotive, and medical, based on their flexibility given to designers to fabricate complex structures, which are hard to fabricate using conventional methods. By achieving lightweight structures that do not require molding and tooling, AM also saves time, cost, and effort. Also, AM plays an important role in hybrid manufacturing and smart factories [2], and it is a key technology in the implementation of the new industrial revolution, Industry 4.0 [3].

AM has a multi-disciplinary characteristic and its standardization is essential for the industrial sector. The main organizations which are working and collaborating to standardize the processes of AM are the International Organization for Standardization, the technical committee ISO/TC 261 (creation date 2011), the American Society for Testing and Materials, the group ASTM F42 (formed in 2009), and the European Committee for Standardization, the technical committee CEN/TC 438 (formed in 2015). ISO/ASTM 52900-15 established and defined terms used in AM technology, by defining AM as the “process of joining materials to make parts from three-dimensional (3D) model data, usually layer upon layer, as opposed to subtractive manufacturing and formative manufacturing methodologies” [4]. Based on the ISO/ASTM 52900-15 standard, the AM processes are classified as follows [4]: vat photo-polymerization (VP), binder jetting (BJ), material extrusion (ME), material jetting (MJ), sheet lamination (SL), powder bed fusion (PBF), and directed energy deposition (DED). Some examples of the technologies that fall within these processes types include stereolithography (SLA) as a VP process [5], fused deposition modeling (FDM) as an ME process [6], laminated object manufacturing (LOM) as an SL process [7], selective laser sintering/melting (SLS/SLM) as a PBF processes [8,9], 3D inkjet printing (3DP) as a BJ process [7], polymer jetting (PolyJet) and multi-jet printing (MJM) as MJ processes [10,11,12], wire and arc additive manufacturing (WAAM) and laser-engineered net shaping (LENS) as DED processes [13]. 

The performance characterization of the AM processes and material characterization are significant tasks for their standardization and implementation in the industry. A method for evaluating of AM process performance consists of manufacturing and testing a customized sample [14]. The most important sections, focusing on test methods and product characterization of the parts manufactured by AM [4], are mechanical properties (hardness, tensile strength, flexural strength, impact strength, fatigue strength, elongation property, compressive properties, creep, aging, frictional coefficient, shear resistant, crack extension), surface aspects (appearance, surface texture [15], color), and geometry requirements [16] (size, dimensions, dimensional tolerances, geometrical tolerance). Currently, the standards related to these items are under development. A number of experimental and theoretical studies were developed in order to characterize the variety of AM processes and the materials used for AM. 

### 1.2. Surface Quality in Additive Manufacturing

The surface quality of the parts built by the AM technologies is an important parameter, which can influence the product accuracy, minimize the post-processing operations, and enhance the functionality of the product. Within the literature, many studies focused on the surface roughness [5,6,8,10,11,12,17,18,19] of the parts made through additive manufacturing. Some studies investigated the influence of part orientation on surface roughness [7,20], for different layer manufacturing technologies such as FDM, SLS, LOM, and SLA. An optimal fabrication direction achieves good results. Canellidis et al. [20], considering the build time, surface roughness, and post-processing time, proposed an optimization procedure for optimum build orientation of the parts in SLA. Perez et al. [6] investigated the critical factors in FDM processes for reducing surface roughness, and they concluded that the layer height and wall thickness are dominant factors for surface roughness. DebRoy at al. [9], analyzing the AM metal processes, showed that surface quality is influenced by the material feedstock, part and process design, process parameters, and post-processing. The surface roughness is different in different AM processes. The main factor that affects the surface roughness in different AM processes is the deposition layer thickness. Also, in metal additive manufacturing, whatever might be the parameters, the surface roughness is high in DED processes because of a higher deposition rate [13] and larger size of melt pool. Thus, it is not fair to compare directed energy deposition with powder bed fusion from the point of view of surface quality.

Additive manufacturing of parts with a small deposition layers thickness reduces the surface roughness and improves the surface quality. PolyJet technology, using thin deposition layers of 16 or 30 microns [12,19], is one of the AM technologies that can effectively reduce the surface roughness. A benchmark study based on customized test specimens investigated and compared the properties of nonmetal additive manufacturing processes (SLA, PolyJet, SLS, LOM, FDM, and 3DP) [17]. It concluded that the roughness of the specimen fabricated by EDEN 500V increases with the surface angle from 0° to 90°, and that PolyJet technology is effectively an office environment. An experimental investigation of the surface roughness of the PolyJet process, in *X*- and *Y*-directions, based on a prismatic test sample placed seven times, on the same platform was performed in Reference [19]. The results indicated that the best value of roughness was achieved at 0° and the worst at 90°. The experiments on an EDEN 350V machine, with samples made of FullCure 720 and VeroBlue 840 materials, oriented along the *x*-axis were carried out in order to investigate the influence of layer thickness, local surface orientation, and finish type on the surface quality [12]; however, different part orientations on the build tray were not performed. This study reported that the maximum surface roughness was obtained for the 90° surface angle. Moore and Williams [21] investigated the effects of the surface finish on the part fatigue life of PolyJet parts. It was concluded that the glossy finish increases the fatigue life of the printed parts. 

Ituarte et al. [22] performed a comparative assessment of three AM technologies (SLA, Polyjet, and SLS) taking four control factors (the AM machine, the part orientation and location, and the chordal errors of the STL files), and having one of the objectives to be optimized (i.e., the surface roughness). The study reported that the manufacturing of flat plastic parts using the three selected AM systems is not technically feasible. The main reason could be that the layer thickness set on a value greater than 30 μm increases the surface roughness. Also, the results showed that surface quality was the most difficult requirement to satisfy.

Most parts obtained by different additive manufacturing technologies need selecting a suitable post-processing technique (computer numerical control machining, abrasive machining, laser machining, chemical treatments, etc.) to improve the surface quality [23]. Cazon et al. [11] included in their studies the effect of post-processing on the mechanical properties and the surface roughness of planar specimens made by the PolyJet technology on the EDEN Objet 330 machine. The study concluded that the part orientation has an effect on mechanical properties and the best roughness is obtained for the *XY* printing direction. An industry-related survey including 50 interviewees evaluated technical and economic AM capabilities [24]. The study concluded that post-processing constitutes a mandatory production step to fulfill minimum surface roughness levels required by the industry.

From the literature survey, the following results were found:

- The surface quality of additive manufactured parts can be affected by different factors, from pre-processing, processing, and post-processing stages.
- The factors affecting the surface quality are STL file preparation, material properties, layer thickness, part orientation, scanning parameters (strategy, speed, etc.), surface orientation or surface angle, finish type, and post-processing.
- AM technologies and variation from one AM machine to another can influence the surface quality.
- The staircase effect, support structure burrs, and errors from the deposition process are the most frequent surface defects.
- There are few research studies developed on different AM systems built based on PolyJet technology (Stratasys Ltd, USA and Objet Geometries Ltd., Israel).
- Having benefits in terms of cost reduction and a shortening of the time-to-market in products, the implementation of AM technologies within pre-production series and short series production depend on the repeatability of mechanical and surface quality properties of parts.

### 1.3. Test Artifacts for Quality Surface Evaluation

Many test artifacts were proposed to investigate the surface quality of the parts obtained by different additive manufacturing processes. A standardized test artifact allows the performance estimation and comparison for different AM systems or for the same AM system over time [14] and can identify the possibilities for improvements. 

**Figure 1:**
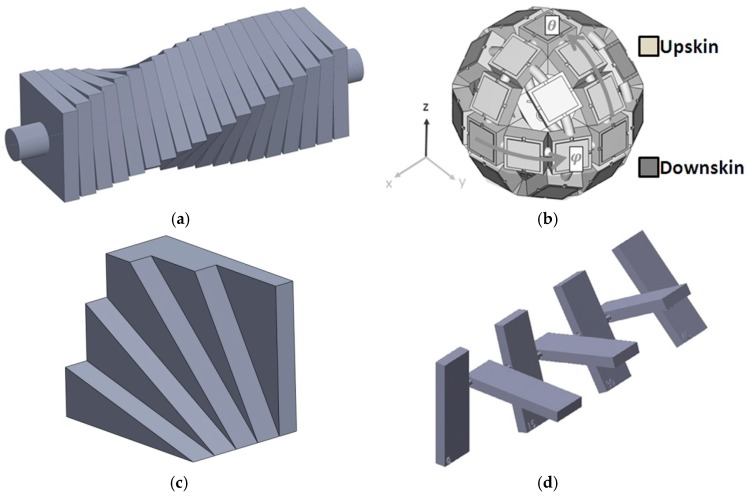
Test artifacts: (**a**) truncheon; (**b**) faceted sphere [26]; (**c**) specimen with sloped surfaces; (**d**) Standard Test Artifact (STAR) [14,26].

A twisted pillar [5,8,12,18,25] or truncheon (Figure 1a) is one of the most used models for measuring the surface roughness within an AM process, despite its massive shape. Thus, it can be used for investigations of the surface roughness based on the truncheon within the following AM technologies: SLA (vat photo-polymerization) [5,18,25], FDM (material extrusion) [5], PolyJet (material jetting) [12], SLM (powder bed fusion) [8], and LOM (sheet lamination) [5]. The twisted pillar is obtained by rotating a series of square sections around a central axis, from 0° to 90°, in 3° or 5° increments, allowing to investigate the surface roughness of angled planes from 0° to 360° [18,26].

Another test model is a faceted sphere (Figure 1b) which covers a wide range of planar surface orientations within one part [26,27]. A revised model with new features, based on a specimen proposed by Kim et al. [17], is shown in Figure 1c. The ISO/ASTM NP 52902 standard focusing on AM standard test artifacts is under development. Some standard artifacts were developed by the ASTM F42/ISO TC 261 joint group for Standard Test Artifacts (STAR) in order to investigate the AM system performances: linear and circular accuracy, resolution of ribs, pins, holes and slots, and surface texture [14]. The STAR group proposed a surface texture artifact [14,26] which consists of a series of seven platens built at different angles to the horizontal plane, including 90°, 0°, 75°, 15°, 60°, 30°, and 45° (Figure 1d). 

The studies focusing on the quality surface investigation obtained by AM did not use a common surface inspection artifact that allowed comparing the measurements. Moreover, a deeper research based on a standardized surface inspection artifact taking into account the relevant build orientations on the manufacturing platform and post-processing operations was not performed.

The main aim of this article was to define a basic methodology that allows evaluating the performances of an AM process and material characterization in terms of surface quality, based on a new test artifact and investigating the primary factors that affect the surface quality. The second part of the paper focuses on the investigation of polymer-based AM. A case study on a PolyJet process validates the proposed basic methodology. 

## 2. Materials and Methods 

### 2.1. Design of a Test Artifact for Evaluating the Quality Surface Performance of AM Systems

The main design requirements of a surface inspection artifact are as follows:

- An easy adaptation to different AM processes and machine sizes;
- Measurements to be easily performed in different ways using contact or non-contact methods;
- An editable geometry;
- A short manufacturing time;
- Minimum material and energy consumption.

A new surface inspection artifact was designed using the SolidWorks version 2013 software (Dassault Systèmes, Waltham, MA, USA), based on the above design requirements. It consisted of rotated platens around a horizontal axis, which were built at different angles from 0° to 90° to the horizontal plane, in θ° increments. Eight design configurations of the test artifact were achieved, taking into account that the increment θ was a divisor of 90° (Table 1).

The platen thickness should be chosen so as not to allow the artifact to deform over time, being limited by the characteristics of the material. The dimensions of the platens (length and width) should be chosen so that the surface roughness can be measured easily with both contact and non-contact methods. 

The artifact was intended to be used to evaluate the surface quality of an additive manufacturing process and to test the machine accuracy. Mainly, the artifact was designed to investigate the surface quality of the upward facing surfaces. The downward facings are strongly dependent on the support structure type, which influences the surface quality. This artifact allows good accessibility for the surface investigation of the platens.

**Figure 2:**
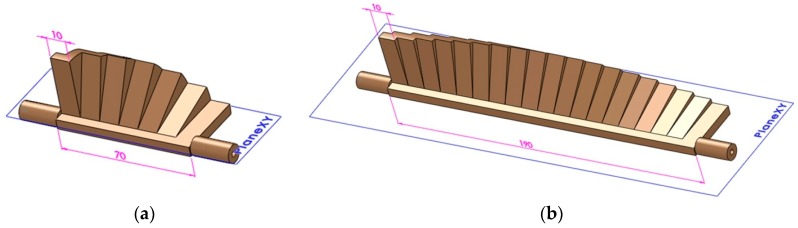
Three-dimensional (3D) models of the new test artifact: (**a**) artifact configuration 3; (**b**) artifact configuration 6.

Two 3D computer-aided design (CAD) examples of the test artifact built at angles from 0° to 90° in steps of 15° and in steps of 5° are shown in Figure 2.

### 2.2. Methodology for Evaluating the Surface Quality in AM

**Figure 3:**
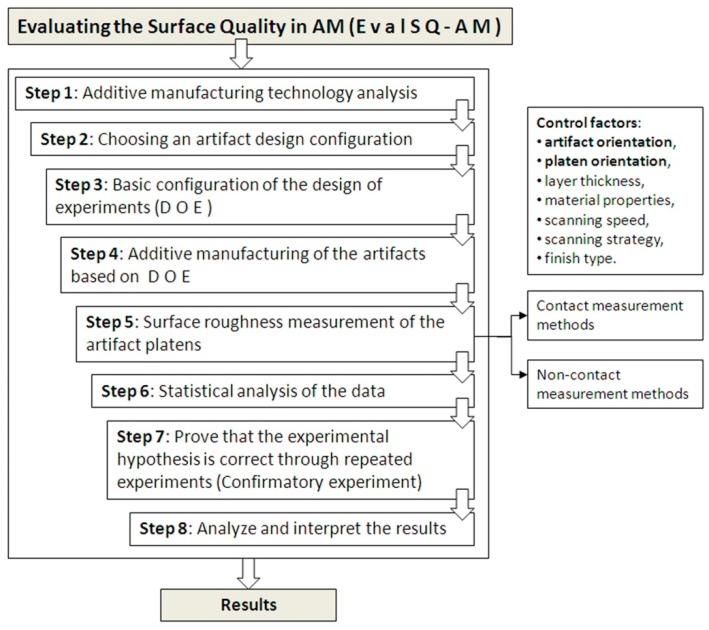
Flowchart of the proposed methodology named EvalSQ-AM.

The objective of the proposed methodology is to evaluate the performance of an additive manufacturing process on an additive manufacturing machine in terms of surface quality. This methodology includes exploratory and confirmatory experiments, followed by analysis and interpretation of the results. The methodology consists of eight steps as shown in the Figure 3.

#### 2.2.1. Step 1: Material and Additive Manufacturing Technology Analysis

There is a large diversity of materials used in additive manufacturing processing [28]: plastics (thermoplastics and thermosets), metals, ceramics, and composites (polymer matrix composites, metal matrix composites, and ceramic matrix composites). Thus, the additive manufacturing technologies include polymer-based AM, metal-based AM, ceramic-based AM, and composite-based AM. The purpose of AM technology analysis is the understanding of the process basics principles and selection of some potential parameters that influence the manufacturing process and can affect the surface quality. 

#### 2.2.2. Step 2: Choosing an Artifact Configuration 

The design configuration of the surface inspection artifact was chosen from Table 1. An artifact configuration was defined by a number of platens in steps of θ° from a 0° to 90° slope angle. Every artifact configuration due to its size and volume influenced the manufacturing time, material consumption, and the amount of measurement data used within step 6 for statistical analysis.

#### 2.2.3. Step 3: Basic Configuration of the Design of Experiments (DOE)

In the third phase of the methodology, based on the additive manufacturing technology features, we designed the experiments [29] by choosing the control factors that affect the surface quality and their levels. The primary control factors that affect the surface quality are the layer thickness, the artifact orientation, the platen orientation, the material feedstock properties, the scan speed, the scanning strategy or tool path strategy, the energy density, and the finish type based on particular printing features or post-processing. The selection of the control factors depends on the particularities of the additive manufacturing technology. The main factors, which should be used for all additive manufacturing technologies, are artifact orientation and platen orientation.

**Figure 4:**
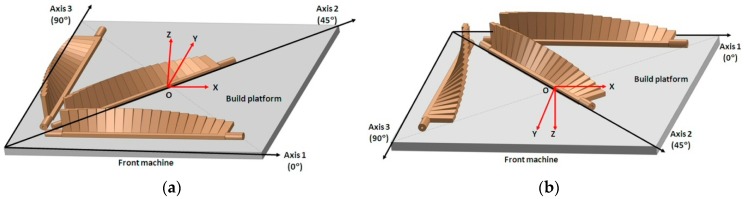
Layout of the build platform illustrating basic artifact orientations for (**a**) upward building additive manufacturing (AM) systems, and (**b**) downward building AM systems

The impact of nesting and orientation of parts that are inserted into the build platform on production speed, material costs, and costs related to process time for different AM process (ME, VP, BJ, MJ, SL, and PBF) was investigated in Reference [30]; however, the surface quality of the parts was not estimated. The study concluded that the most affordable way of manufacturing parts does not guarantee constant or similar quality. The orientation of the AM build platform coordinate system depends on building direction. Thus, the coordinate systems of downward and upward building machines are defined based on the standard in Reference [31] and are shown in the Figure 4. The artifact orientation on the build platform is considered a three-level factor with basic orientations of parallel, perpendicular, and 45° to the scanning direction (called axes 1, 2, and 3, respectively). The layout of these three artifact orientations within the build platform is called the basic artifact orientations. The levels of platen orientation factor depend on the design configuration of the artifact, as shown in Table 1. It is given by the number of platens of the artifact.

The layer thickness factor depends on the characteristics of the AM technology. Also, this control factor strongly influences the surface quality. The feedstock material for additive manufacturing can be a liquid, powder, combination of powder and wire, sheet, or wire, according to the process in question [13,28]. The feedstock materials can affect the surface quality. Regarding the scan speed factor, only some AM technologies allow modifications to its values. Thus, one can mention powder bed fusion and directed energy deposition technologies, which allow the variation of the scanning speed factor. Depending on the additive manufacturing technology characteristics, some AM technologies allow modifications in the scan strategy and others do not. The scan strategy factor is taken into consideration mainly in powder bed fusion [32] and directed energy deposition [33] technologies. The tool path strategy factor is important in material extrusion technologies [6]. The energy density is a specific factor within metal additive manufacturing technologies that needs to be carefully controlled [9]. The two basic control factors, artifact orientation and platen orientation, and additional process factors should be chosen to analyze the influence of their effects and interactions on the surface roughness.

#### 2.2.4. Step 4: Additive Manufacturing of the Artifacts Based on DOE

Additive manufacturing of the artifacts based on DOE represents the fourth phase of this procedure. The basic table configuration is shown in the Figure 4. A minimum three artifacts oriented in a 0°–45°–90° configuration should be 3D-printed in a single printing job. In the pre-processing stage, the CAD model of the test artifact is converted into a standard triangulation language (STL) file and is loaded into specific AM software in order to manage the manufacturing process. The STL resolution should be chosen according to the deposited layer thickness of the AM machine. The post-processing method depends on the type of AM technology and raw material used [23].

#### 2.2.5. Step 5: Surface Roughness Measurement of the Artifact Platens

The surface roughness measurements may be performed in different ways using contact or non-contact methods. Only the surface of up-facing platens is measured, because the down-facing platens are strongly influenced by the support material.

#### 2.2.6. Step 6: Statistical Analysis of the Data

Statistical analysis of the data allows the investigation and characterization of the effects of control factors and their interactions on the experimental surface roughness. Because there are two or more control factors, two-way ANOVA or generalized linear models (GLM) should be used. The generalized linear model is a more general approach to performing an analysis of variance (ANOVA) [34]. The statistical analysis should have five stages as follows:

- Inputting data;
- Performing data analysis;
- Determination of significant factors from ANOVA table;
- Validating ANOVA assumptions;
- Interpreting ANOVA results.

GLM is used to analyze the results by calculating the means of each level for each factor. From the ANOVA table, the *p*-value indicates the significance of the results. Additionally, it is necessary to compare the relative significance by indicated the *F*-value when the *p*-value is close to zero. Before drawing the conclusion, the three following assumptions in ANOVA analysis should be checked [34]:

- Residuals are normal distributed;
- The variance of the observations in each treatment should be equal;
- Response is independent and identically distributed.

#### 2.2.7. Step 7: Prove that the Experimental Hypothesis Is Correct through Repeated Experiments

The confirmatory experiments should be conducted in an environment as similar as possible to the original experiment. An industrial process is desirable for stability. The purpose of the confirmation experiment is to validate the conclusions drawn during the analysis phase and to confirm having a stable process.

#### 2.2.8. Step 8: Analyze and Interpret the Results

The experimental results are analyzed and interpreted within this step. The roughness prediction for an AM process and the influence of the control factors and their interactions on the surface roughness are determined.

### 2.3. Methodology Application—PolyJet Material Jetting Technology

The proposed methodology for evaluating the surface quality was validated for a polymer-based additive manufacturing technology. The additive manufacturing machine used was EDEN 350 by Objet (Stratasys, Rehovot, Israel ) using PolyJet technology.

#### 2.3.1. Materials and PolyJet Technology Analysis

**Figure 5:**
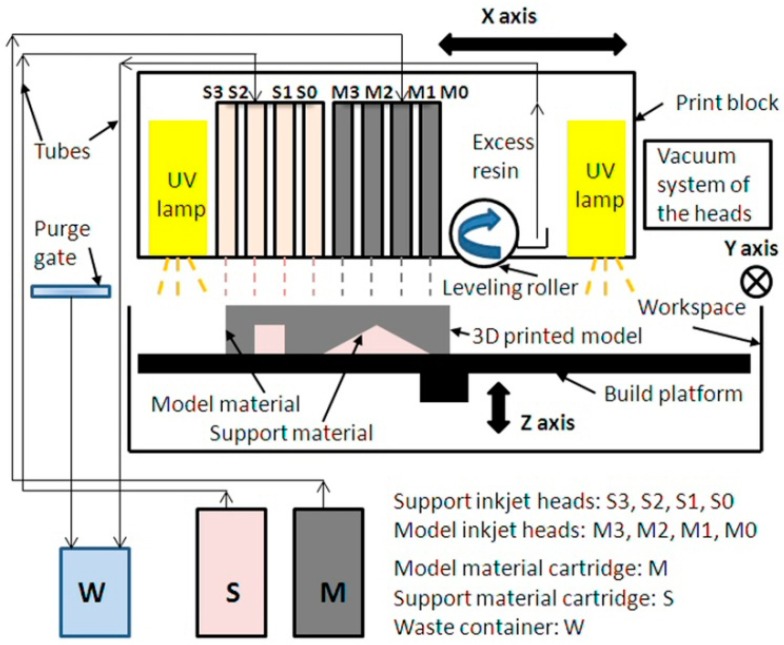
The basic principle of the Objet EDEN 350 PolyJet machine.

The Objet EDEN 350 PolyJet machine uses a drop-on-demand (DOD) inkjet technology [35] to generate and selectively deposit droplets of resins in the scanning direction through one pass onto the build platform. The deposited layers of resins are 0.016 mm thick. The print block contains eight parallel print heads, four of which are allocated to the model material and the others to the support material. The model material used in this investigation was FullCure 870 VeroBlack [36], supplied by Stratasys. The composition of the model material consisted of acrylic monomer, urethane acrylate oligomer, epoxy acrylate, and a photo-initiator. The FullCure 705 [36] was used as a support material. The support material consisted of acrylic monomer, polyethylene glycol 400, propane-1,2-diol, glycerol, and a photo-initiator. During the printing process, the print heads and the photopolymer resins are heated at a printing temperature of around 72 °C, decreasing the viscosity of the resins. The print block moves pass by pass in indexing the *y*-axis and layer to layer in the height direction (*Z*). A vacuum system kept and controlled the resins within the print heads (Figure 5). The head vacuum of the Objet EDEN 350 PolyJet machine was set at 6.2 atm. Roller levels each deposit a resin layer and then the resin is hardened using two ultraviolet (UV) lights. The roller compresses the resin layers and removes excess resin material from the deposited layer. The excess resin is stored into a waste container. Lines of droplets are formed in the scanning direction.

#### 2.3.2. The Basic Configuration of the Design of Experiments (DOE)

Artifact configuration 3 (Figure 2b), consisting of 19 platens built at angles from 0° to 90° in steps of 5°, was chosen to investigate the effect of platen orientation on the surface roughness. 

The design of experiments was made according to the control factors. Three control factors were selected (i.e., the artifact orientation with three levels, the platen orientation with 19 levels, and the finish type with two levels), as shown in Table 2. 

**Figure 6:**
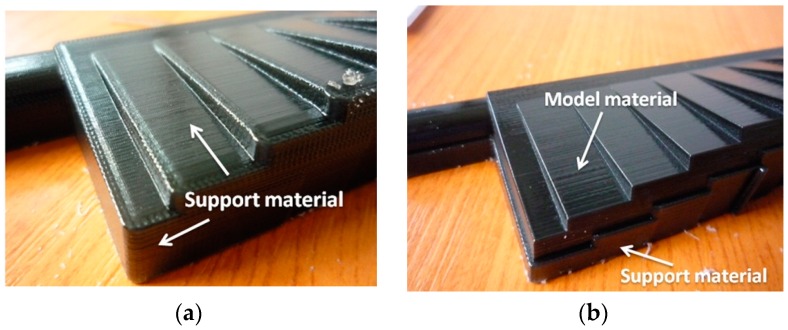
Finish type within PolyJet technology: (**a**) matte finish; (**b**) glossy finish.

The finish type was considered as a two-level factor: matte and glossy. The matte finish (Figure 6a) meant that a thin layer of support material surrounded the part. When using a glossy finish (Figure 6b), there was no support material deposited on the upper surfaces of the model; instead, it was only deposited on the bottom surfaces. The points of transition between the matte and glossy areas usually required some polishing or extra cleaning. 

The working parameters were set according to the manufacturer’s specifications as shown in Table 3. The surface roughness was the response factor during the experiments. 

It is necessary to specify why only three control factors were chosen. Some parameters of this Objet EDEN 350 PolyJet machine cannot be modified, being set by the provider. These are scanning speed, scanning strategy, and layer thickness. These factors should be taken into consideration for other AM processes, which allow modification in layer deposition thickness or scanning speed. However, Polyjet technology used on the EDEN 350 machine allows only deposition of thinner layers of 0.016 mm. In addition, Chen [10] concluded that, for very low thickness, the effect of change in layer thickness has a very low contribution toward change in surface roughness. 

The experiments and measurements were performed under controlled laboratory temperature and relative humidity, as shown in Table 3.

#### 2.3.3. Additive Manufacturing by PolyJet of the Artifacts Based on DOE

The software used to prepare the build platform for producing models and to simulate the build time and material consumption was the Objet Studio version 8.0.1.3 software (Rehovot, Israel) [36]. 

**Figure 7:**
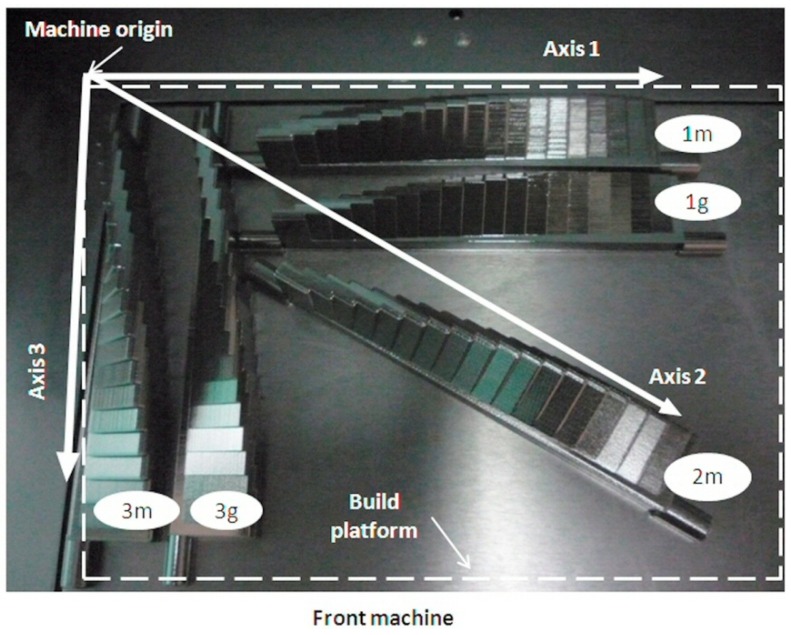
Layout of the EDEN 350 build platform illustrating the artifact samples in three different orientations.

Five test artifacts, named as in Table 4, were manufactured on an Objet EDEN 350 PolyJet machine (Stratasys) in the same printing job (using a single build platform) from FullCure 870 VeroBlack model material (Figure 7). The support material was removed with a pressure water jet. The specimens were immersed in a sodium hydroxide solution and were washed with pressurized water afterward.

In material jetting technology, some rules should be applied regarding the orientation of the part and parts within the build platform in order to minimize the manufacturing time and material consumption, and to improve the quality of the surface [37]. The test artifacts were built in three different orientations (Figure 7), namely the longest dimension parallel to the scanning direction (Axis 1), perpendicular to the scanning direction (Axis 2), and 45° to both directions (Axis 3). The samples oriented in Axis 1 and Axis 3 were fabricated in high-quality mode, either with a matte or glossy finish. The sample aligned with Axis 2 was 3D-printed in high-quality mode with a matte finish. A glossy sample aligned with Axis 2 was not chosen to be fabricated because the glossy finish applied to an unaligned sample to the printer’s axes can generate surface flaws, especially if the sample has “straight-line” walls [37]. 

#### 2.3.4. Surface Roughness Measurements of the Artifact Platens

**Figure 8:**
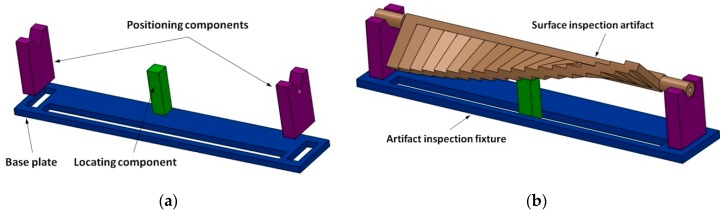
(**a**) The components of the artifact inspection fixture; (**b**) positioning of the artifact on the fixture.

A modular artifact inspection fixture was designed to easily investigate the artifact platens. This fixture consisted of four flat components, as shown in Figure 8a: a base plate, a locating component, and two positioning components. Taking into account the post-processing steps [23] and costs of AM processes for the main categories of materials (plastics, metals, and ceramics), the new fixture is proposed to be manufactured particularly in the case of a polymer-based AM process.

The components of the artifact inspection fixture were built on the Objet EDEN 350 PolyJet machine in the same printing job from FullCure 720 material. All these components were built with a glossy finish. The test artifact was positioned on the fixture, based on its cylindrical surfaces and the locating component that slides in the slot of the plate, as seen in Figure 8b.

**Figure 9:**
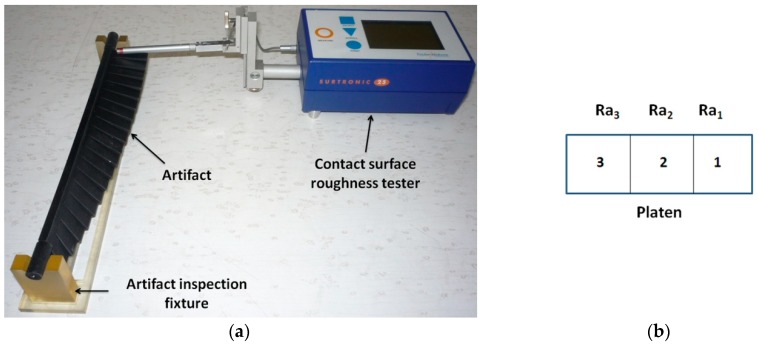
(**a**) Surface roughness measurement of the up-facing surfaces; (**b**) details of the measurement procedure.

Jouini et al. (2009) [38] carried out a multi-scale analysis of high-precision surfaces using three different techniques: stylus profiler, scanning white-light interferometry, and atomic force microscopy. It was concluded that the stylus profiler is optimal for the study of macro-roughness surfaces. Each specimen was measured using a Surtronic 25 contact surface roughness tester (Hoofddorp, The Netherlands) from Taylor Hobson, as per the DIN EN ISO 4288 standard [39], as shown in Figure 9a. This is a portable and flexible roughness checker that, technically, makes the roughness assessment feasible for this research. The surface roughness checker was calibrated before performing the measurements. Usually, an evaluation length equal to five consecutive cut-off lengths provides a good result [39]. The standard DIN EN ISO 4288 provides information for conventional manufacturing processes. Moreover, guidelines on how to use the standard for AM component measurement should be provided. The Gauss-filtered measurements were set up for an evaluation length of 4 mm and a cutoff value of 0.8 mm for the artifacts printed in a glossy finish, and an evaluation length of 12.5 mm and a cutoff value of 2.5 mm for the artifacts printed in a matte finish. 

The surface roughness, Ra (the arithmetic mean deviation), was evaluated. Three measurements were performed for each platen orientation of the artifact specimens. Thus, each platen was divided into three areas, as shown in Figure 9b. The measurements of Ra1, Ra2, and Ra3 were taken in areas 1, 2, and 3, respectively. Therefore, 57 measurements were taken for each artifact, obtaining 285 measurements for the experiment. The same number of measurements was performed for the confirmatory experiment. Finally, the average roughness Ra was calculated for each platen based on the three surface roughness measurements.

In order to evaluate the measurement system and to see if it was acceptable, the variability caused by the roughness measurement device was investigated to understand how large the variability of the measurement system was in comparison with the part variation. As only one appraiser was used, three measurements were made for the 19 platens of the artifact. The data were processed with the support of Minitab 17 software (Coventry, UK) using Gage R&R study and the interpretation of the data was based on Reference [40].

#### 2.3.5. Statistical Analysis of the Data

The purpose of the statistical analysis is to analyze the effects of different factors and their interaction on the surface roughness measurements. A general full factorial design with 114 factor combinations was performed to be able to investigate the influence of the artifact orientation, the platen orientation, and the finish type on the surface roughness. The contribution of the three factors to the surface roughness was determined using the generalized linear model (GLM) analysis within the Minitab 17 software [41]. Based on generalized linear model analysis, the contribution of the factors and their two-way interactions was calculated by selecting the sequential sum of square (Type I). The *F*-values and the *p*-values were analyzed in order to make a decision concerning the statistical significance. It is necessary to compare the relative significance by indicating the experimental and critical *F*-values when the *p*-value is close to zero. The critical value is the number that the test statistic must exceed to reject the test. The percentage contribution ratio (PC%) of each factor and interactions was calculated by dividing each sequential sum of squares (Seq SS) by the total sequential sum of squares and multiplying by 100. This parameter shows the significance of the effect of each factor and their interactions.

The confirmatory experiment was performed in similar conditions as the initial experiment (temperature, humidity, Objet EDEN 350 PolyJet machine’s parameters). Also, it used the same raw materials. Based on generalized linear model analysis, the contribution of the factors and their two-way interactions was calculated for the confirmatory experiment. 

## 3. Results and Discussions

The results of the proposed methodology for the PolyJet process on the Objet EDEN 350 PolyJet machine were analyzed taking into account the following factors:

- The experimental surface roughness distribution of up-facing platens;
- Comparison of the experimental surface roughness with theoretical models;
- Surface quality issues of PolyJet samples;
- Results of statistical analysis.

**Figure 10:**
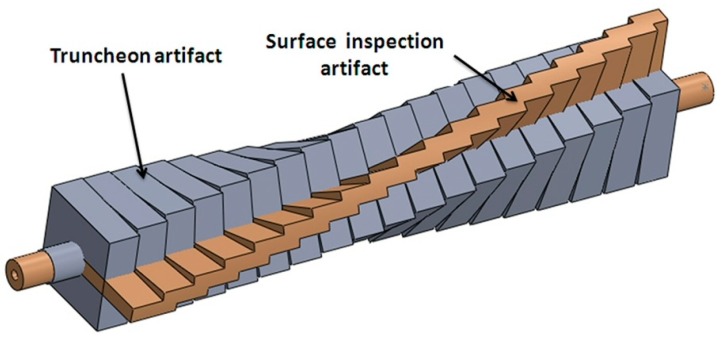
Comparison between the new test artifact and the truncheon artifact with the same dimensions of the flat surface to be measured.

All five artifacts were manufactured in 10 h and 42 min, using 452 g of model material and 414 g of support material. The processing time for the components of the artifact inspection fixture was 46 min, and the consumption was 99 g of model material and 24 g of support material. A comparative study carried out by overlapping the CAD models of the proposed artifact and the truncheon artifact, with the same dimensions of the flat surface to be measured, is shown in Figure 10. The new test artifact allowed the model material consumption to be reduced by 83% compared to the truncheon which is used for roughness investigation in most AM technologies.

### 3.1. The Experimental Surface Roughness Distribution of Up-Facing Platens

Surface roughness results determined from experiments were analyzed to predict their distribution. A detailed prediction of the experimental roughness is shown in Table 5. 

**Figure 11:**
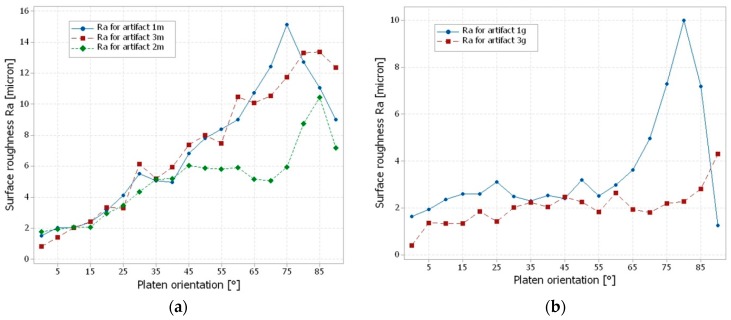
Trends of the measured roughness (Ra): (**a**) artifacts printed in matte finish type; (**b**) artifacts printed in glossy finish type.

The experimental roughness (Ra) values of the artifacts printed in matte finish were low in the range of 0.5–4 μm for a domain of 0–25° platen orientation, increasing to maximum values of 10–15 μm for the platen orientation of 75–85° and then decreasing for the 90° surface angle, as shown in Figure 11a. The roughness of the artifacts printed in glossy finish presented different trends (Figure 11b). Specifically, the roughness of the 1g specimen showed a constant trend in the domain of 0–65° platen orientation, increasing until 80° and then decreasing at 90°. The Ra roughness of the 3g specimen showed a constant trend in the domain of 0–80° platen orientation and then increased at 90°.

### 3.2. Comparison of the Experimental Surface Roughness with Theoretical Models

Theoretical predictions of surface roughness (Ra) depending on the surface angle were developed by many authors [5,12], considering different AM techniques. Within additive manufacturing, the theoretical studies estimated the Ra surface roughness, based on trigonometry [25], interpolation of measured data [18], a database of roughness values experimentally determined analyzed by specific algorithms, and intelligent methodology based on genetic algorithms for optimization of the build orientation [5]. The research studies [5,18,25] made on layer manufacturing technologies such as SLA, FDM, and LOM show that, generally, the up-facing surface roughness decreases from a maximum value obtained for a surface angle between 0° and 4° to a minimum value reached around 90°. A theoretical model for material jetting, including the PolyJet technology, should take into consideration the droplet impact with the built platform. Kumar et al. [12] determined a theoretical model for PolyJet technology in the case of matte finish, described by Equation (1). They determined an optimal contact angle of 12° of droplets for the Vero Blue 840 resin.

(1) $$R_{a}=\frac{t}{4}\cdot \left|\frac{\sin\left(\theta +\psi \right)}{\sin\left(\psi \right)}\right|.$$

A theoretical model taking into accord the post-processing is proposed in Equation (2). It estimates the surface roughness for the PolyJet technology in matte build style. The parameters of Equations (1) and (2) are layer thickness (t), platen orientation (θ), droplet contact angle (ψ), and post-processing correction coefficient (K_1_). The correction coefficient K_1_ is less than one, indicating the influence of post-processing on the surface roughness.

(2) $$R_{a}=\frac{t}{4}\cdot \left|\cot\psi \sin\theta +\cos\theta \right|\cdot K_{1},\;K_{1}<1.$$

**Figure 12:**
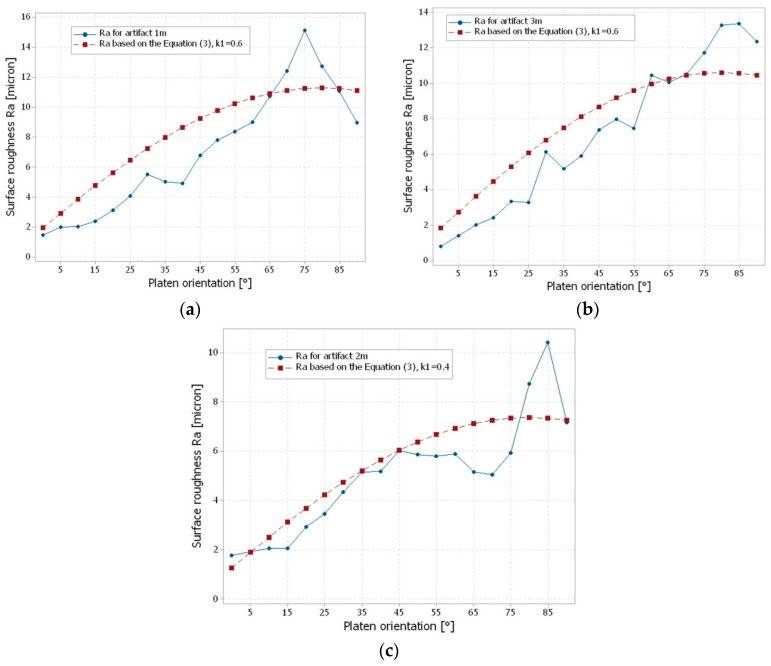
Comparison of experimental roughness and simulated roughness based on Equation (2), for matte artifacts aligned with (**a**) Axis 1, (**b**) Axis 2, and (**c**) Axis 3.

The theoretical curves of the surface roughness distribution (Figure 12), determined by Equation (2), were compared with the experimental roughness curves. An optimization study of the correction coefficient K_1_ was carried out, for all artifact orientations, in the case of matte finish type. The values of the correction coefficient K_1_, which gave the lowest standard mean error, are shown in Table 6.

### 3.3. Surface Quality Issues of PolyJet Samples

Chen et al. [10] analyzed the surface quality of the cylindrical parts printed on the Objet EDEN 350 and observed some errors (rough surface areas) on a layer perpendicular to the scanning direction. These were caused by the lower resolution of 600 dots per inch (DPI) (0.042 mm) in the *X*-direction and *Y*-direction compared to 1600 DPI (0.016 mm) in the *Z*-direction. 

**Figure 13:**
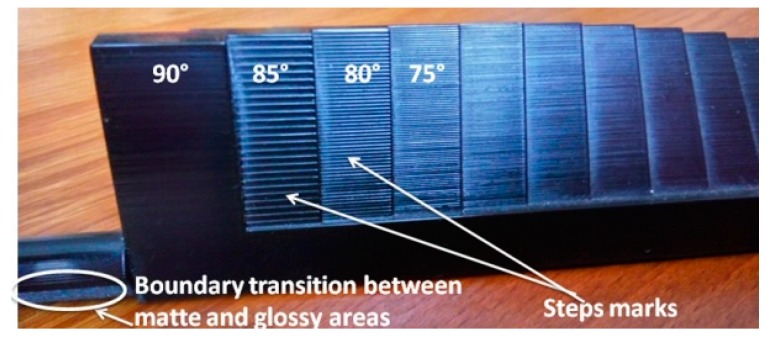
Flaws for samples printed in glossy style involve steps marks and a boundary transition between matte and glossy areas.

Some steps marks (Figure 13) were visually observed on the artifacts printed in the glossy mode, for the platen orientation starting from 75° to 85°. The density of marks on the platens decreased with the increase in angle; however, it increased their height. The highest marks were observed for 85°. The vertical walls printed in the glossy style had excellent surface quality without any visual flaw. A glossy–matte boundary flaw (Figure 13) was detected on the cylindrical feature printed in the glossy mode. The glossy–matte boundary delimited the upper surface printed in the glossy mode and the lower surface affected by the support material.

### 3.4. Results of Statistical Analysis

Base on Reference [40], the results of the Gage R&R study showed that the measurement system is acceptable if the percentage of variance components is less than 1%. This condition was satisfied for each artifact measured, with repeatability proven, whereby the roughness device variation was much smaller than the variation of the surface roughness of the parts manufactured by the AM process, as shown in Table 7. In this study, only one appraiser recorded the data, making reproducibility unnecessary.

#### 3.4.1. Determination of Significant Factors: ANOVA table

The results are listed in Table 8. From the ANOVA table, it can be seen that the platen orientation and the finish type had a higher significant influence on the Ra, as long as the *p*-value was lower than the significance level 0.05 (even lower than 0.001). Also, the factors platen orientation, finish type, and their interaction showed *F*_exp_ values greater than the critical *F*-value of 0.1% at α = 0.001. It can be concluded that the results were significant at the 0.1% significance level.

The most significant percentage contribution ratios were obtained for platen orientation, finish type, and their interaction. The most significant factor on the roughness parameter (Ra) was the platen orientation, which explained 46.65% of the total variation. The next largest contribution on Ra came from the finish type, with a contribution of 29.50%. This interaction had a significant effect on Ra, whereby the *F*-value equal to 202.65 was larger than F_0.1%_ (equal to 15.08). The artifact orientation had no important effect on Ra, with a PC% around 0.81% and *F*_exp_ equal to 18.55. The interaction of platen orientation with finish type had a significant effect on the roughness parameter, whereby PC% was 14.50% and *F*_exp_ was equal to 5.53. The interaction of artifact orientation with platen orientation had no significant effect on the roughness parameter (*p* > 0.05).

#### 3.4.2. Validating ANOVA Assumptions

**Figure 14:**
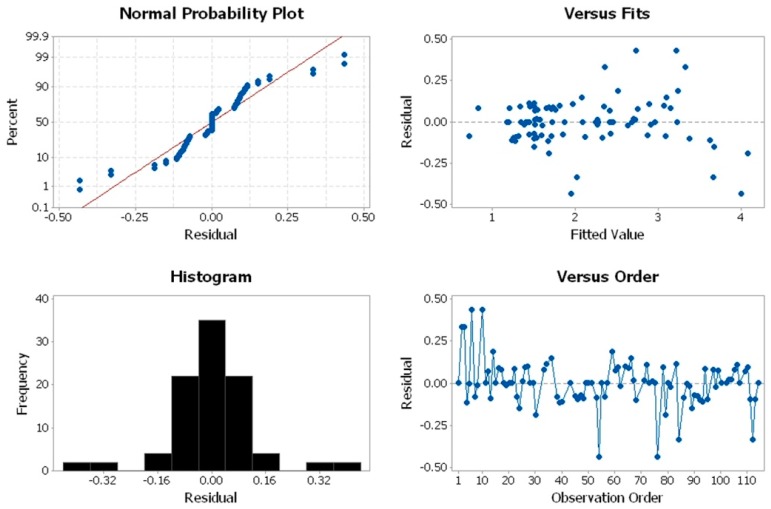
Residual plot for surface roughness (Ra).

The GLM conducted in this section was checked for model adequacy using normal probability plots of residuals, versus fits, and histograms [42]. As can be seen in the normal probability plot (Figure 14), the normality of residual plots was proven as the residuals were normally distributed with two outliers, and the plot resembled a straight line. No common patterns were detected in the graph with residual versus fitted values. Eventually, the independence assumption was satisfied by checking the patterns in the graph of residuals versus orders, whereby no positive correlation or negative correlation was detected.

#### 3.4.3. Evaluation of the Influence of Control Factors on Surface Roughness through Graphical Analysis: Interpreting ANOVA Results

Graphical methods were used to evaluate the influence of control factors on surface roughness. The statistical results were plotted, resulting in the following graphs: the main effects plot, interaction effects plot, and interval plot of Ra versus factor (interval plot of Ra versus artifact orientation, interval plot of Ra versus platen orientation, and interval plot of Ra versus finish type).

**Figure 15:**
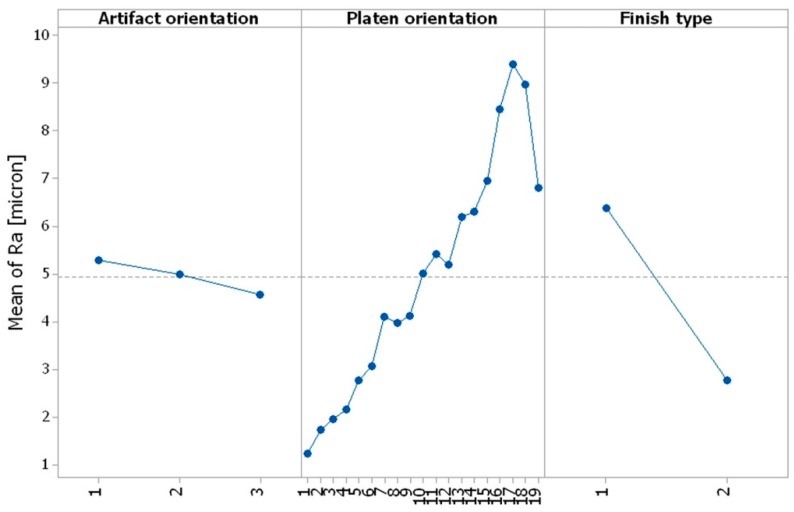
Main effects plot for surface roughness Ra.

**Figure 16:**
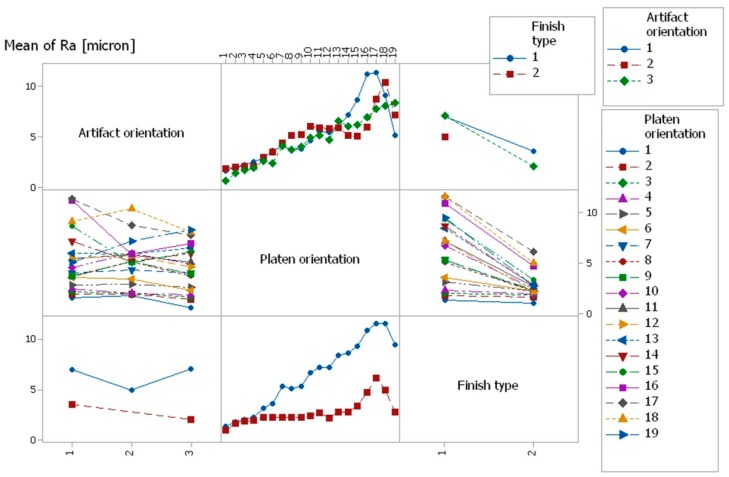
Interaction effects plot for surface roughness Ra.

From Figure 15, it can be seen that the main effects for surface roughness were the artifact orientation at level 1 (0°), the platen orientation at level 17 (80°), and the finish type at level 1 (matte). It is obvious that the factors platen orientation, finish type, and their interaction had a significant influence on the surface roughness, as shown in Figure 15 and Figure 16. 

**Figure 17:**
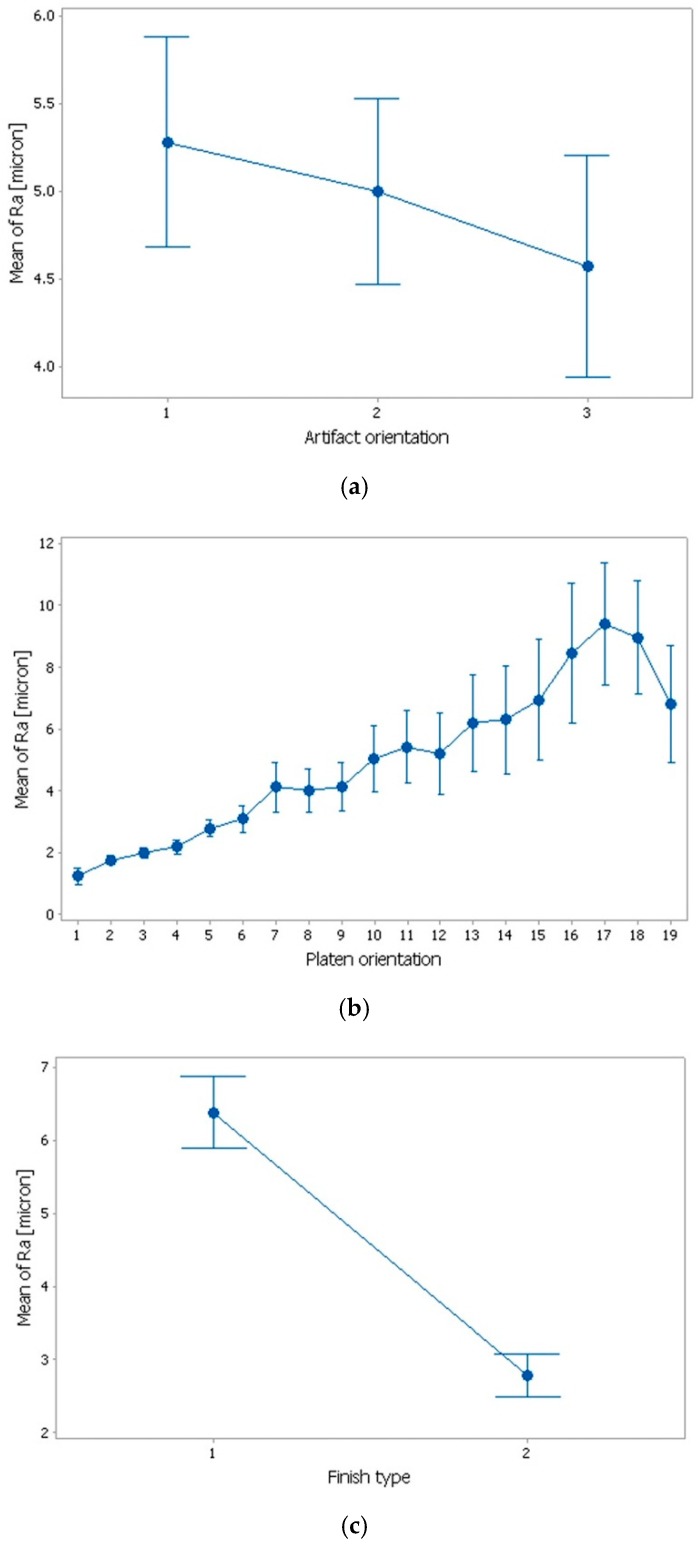
Individual standard deviations were used to calculate the intervals plot of surface roughness (Ra) versus (**a**) artifact orientation, (**b**) platen orientation, and (**c**) finish type. Bars are standard errors of the mean.

The graphs from Figure 17 show the interval plots with standard error bars of each factor versus the roughness (Ra). While the means appear to be different, the difference for Ra in the artifact orientation was probably not significant because all the interval bars easily overlapped (Figure 17a). The platen orientation (Figure 17b) had an influence on the surface roughness and it seems that, at the platen orientation 0°, the mean of Ra was lower, while, for the platen orientation 80°, the mean was higher. There is a possibility to reduce the overlapping if the number of measurements increases, resulting in the difference of the means of the platen orientation at 40° to 80° to be statistically significant. The graph from Figure 17c shows that the difference between the means for Ra in the finish type was significant because the interval bars did not overlap.

#### 3.4.4. Results of Confirmatory Experiment

The differences of means were proven again with high significance for the platen orientation and finish type. 

Since *F*_exp_ was greater than *F*_0.1%_ for the factors platen orientation, finish type, and their interaction, all these results were highly significant at the 0.1% significance level. Moreover, the *p*-value for Ra in the analysis of variance of the artifact orientation (*p* = 0.113) led to the same conclusion as from the first run of the experiments (i.e., probably not significant) (Table 9).

## 4. Conclusions 

This paper investigated the performance of a material jetting AM process and characterized AM materials in terms of surface quality based on the proposed methodology. The following conclusions can be drawn:

- There is a need for surface quality for additive manufactured parts in the industry.
- The implementation of AM for pre-production series and short series production depends on the repeatable surface roughness properties of parts.
- Practical researches as a response to possible industrial beneficiaries need to be performed.
- A family of artifacts was defined to fit different AM system sizes and to meet the needs of a particular customer. Using parametric design techniques, eight different design configurations of the artifact were proposed.
- One of the main advantages of the new artifact over other massive artifacts is its lower volume, resulting in less consumption of materials. Thus, by depositing a smaller amount of material, the AM machine consumes less energy, resulting in lower production costs.
- The shape of the artifact is as simple as possible to ease the process of the build when it comes to time and materials. Also, the simplicity of the artifact shape with flat platens helps in reducing human error when taking measurements.
- The basic artifact orientations allow characterizing the quality surface performance of an AM system in three different basic directions. This can help users and buyers of AM systems to determine how the surface quality depends on the manufacturing direction.
- The flat shape of the platens allows for repeatable measurements and good accessibility for surface investigation.
- The proposed methodology may assess the surface quality of the additive manufacturing process and test the precision of the AM machines.
- The experimental roughness (Ra) values for the PolyJet material jetting process with matte finish were in the range of 0.5–15 μm, with the maximum value for the platen orientation 75–85° and a decreasing trend to 7 μm for 90°. The artifact printed in the glossy finish, oriented perpendicular to the scanning direction, gave the best results in terms of roughness in the range of 0.5–4 μm.
- The drawbacks of the glossy finish include some surface quality issues, such as steps marks at a platen orientation between 75° and 85°, and a boundary transition between the matte and glossy areas.
- From the investigation, the most influential factors on surface roughness for the PolyJet process were platen orientation, finish type, and their interaction.

By carefully choosing the design configuration of the surface inspection artifact and selecting the control factors that affect the surface quality, an efficient design of experiment can be created from the proposed methodology that is applicable to other AM processes using different types of materials. Thus, the methodology can be used to evaluate and compare the performance of the same AM machine in time or between different AM systems. 

## Figures and Tables

**Table 1:** The design configurations of the surface inspection artifact.

Artifact Design Configurations	θ Increment Angle (°)	Number of Platens
Artifact configuration 1	2	46
Artifact configuration 2	3	31
Artifact configuration 3	5	19
Artifact configuration 4	6	16
Artifact configuration 5	10	10
Artifact configuration 6	15	7
Artifact configuration 7	30	4
Artifact configuration 8	45	3

**Table 2:** Control factors and their level.

Level	Artifact Orientation		Platen Orientation		Finish Type	
	Symbol	Value (°)	Symbol	Value (°)	Symbol	Value
1	1	0	1	0	1	Matte
2	2	45	2	5	2	Glossy
3	3	90	3	10	-	-
4	-	-	4	15	-	-
5	-	-	5	20	-	-
6	-	-	6	25	-	-
7	-	-	7	30	-	-
8	-	-	8	35	-	-
9	-	-	9	40	-	-
10	-	-	10	45	-	-
11	-	-	11	50	-	-
12	-	-	12	55	-	-
13	-	-	13	60	-	-
14	-	-	14	65	-	-
15	-	-	15	70	-	-
16	-	-	16	75	-	-
17	-	-	17	80	-	-
18	-	-	18	85	-	-
19	-	-	19	90	-	-

**Table 3:** Working conditions of EDEN 350 machine.

PolyJet Process Parameters	Value
Deposition layer thickness	0.016 mm
System vacuum level	6.2 atm
Temperature of each support head	72 °C
Block temperature behind support heads	72 °C
Block temperature in front of support heads	72 °C
Temperature of each model head	72 °C
Block temperature behind model heads	72 °C
Block temperature in front of model heads	72 °C
Pre-heating of support and model material	68 °C
Chamber temperature	20 °C
Relative humidity	30%

**Table 4:** Printed artifact symbolization.

Artifact Symbolization	Artifact Orientations (°)	Finish Type
1m	0	Matte
1g	0	Glossy
2m	45	Matte
3m	90	Matte
3g	90	Glossy

**Table 5:** A detailed prediction of the experimental surface roughness (Ra).

Finish Type	Artifact Orientation (°)	Platen Orientation (°)	Trends of Experimental Ra (µm)
Matte	0	0–75	an increasing trend from 1.5 to 15
-	-	75–90	a decreasing trend to 9
-	45	0–45	an increasing trend from 1.8 to 6
-	-	45–75	a constant trend around 6
-	-	75–85	an accelerated trend to 10
-	-	85–90	a decreasing trend to 7
-	90	0–85	an increasing trend from 0.8 to 13
-	-	85–90	a low decreasing trend to 12
Glossy	0	0–65	a constant trend with low variation in the range of 2 to 4
-	-	65–80	an accelerated increasing trend to 10
-	-	80–90	an accelerated decreasing trend to 1.3
-	90	0–80	a low variation around the value of 2
-	-	80–90	a low increasing trend to 4

**Table 6:** Theoretical estimation of correction coefficient K_1_.

Artifact Orientation (°)	K_1_	Standard Deviation (µm)	Standard Error Mean (µm)
0	0.6	1.87	0.43
45	0.4	1.69	0.27
90	0.6	1.19	0.27

**Table 7:** The percentage contribution of variance components/artifacts.

Source	Artifact 1m	Artifact 2m	Artifact 3m	Artifact 1g	Artifact 2g
Repeatability	0.33%	0.69%	0.53%	0.56%	0.47%
Part-to-part	99.67%	99.31%	99.47%	99.44%	99.53%
Total variation	100.00%	100.00%	100.00%	100.00%	100.00%

**Table 8:** The percentage contribution ratio based on generalized linear model (GLM).

Source	DF	Seq SS	Seq MS	*F*_exp_	*F*_0.1%_	*p*	PC (%)
Artifact orientation	2	9.66	4.828	2.79	10,15	0.086	0.81%
Platen orientation	18	554.13	30.785	17.81	4.52	<0.001	46.65%
Finish type	1	350.36	350.365	202.65	15.08	<0.001	29.50%
Artifact orientation × platen orientation	36	68.47	1.902	1.10	4.04	0.424	5.77%
Platen orientation × finish type	18	172.18	9.565	5.53	4.52	<0.001	14.49%
Error	19	32.85	1.729	-	-	-	2.76%
Total	94	1187.64	-	-	-	-	100%

**Table 9:** The percentage contribution ratio based on GLM.

Source	DF	Seq SS	Seq MS	*F*_exp_	*F*_0.1%_	*p*	PC (%)
Artifact orientation	2	8.04	4.019	2.45	10,15	0.113	0.67%
Platen orientation	18	561.54	31.197	19.01	4.52	<0.001	46.94%
Finish type	1	352.90	352.903	215.00	15.08	<0.001	29.50%
Artifact orientation × platen orientation	36	70.17	1.949	1.19	4.04	0.352	5.86%
Platen orientation × finish type	18	172.26	9.570	5.83	4.52	<0.001	14.40%
Error	19	31.19	1.641	-	-	-	2.60%
Total	94	1196.10	-	-	-	-	100.0%
